# Clinical Presentation of the SARS-CoV-2 Virus Infection and Predictive Validity of the PCR Test in Primary Health Care Worker Patients of the Spanish National Health System

**DOI:** 10.3390/jcm11010243

**Published:** 2022-01-04

**Authors:** Esperanza Romero-Rodríguez, Luis A. Pérula-de Torres, Jesús González-Lama, Celia Jiménez-García, Rafael A. Castro-Jiménez, Jerónimo J. González-Bernal, Paula Rodríguez-Fernández, Juan Mielgo-Ayuso, Mirian Santamaría-Peláez, Josefa González-Santos

**Affiliations:** 1 Maimonides Institute for Biomedical Research of Córdoba, Reina Sofía University Hospital, University of Córdoba, 14011 Cordoba, Spain; langel.perula.sspa@juntadeandalucia.es (L.A.P.-d.T.); jegonla@telefonica.net (J.G.-L.); celia.jimenez.sspa@juntadeandalucia.es (C.J.-G.); rangel.castro.sspa@juntadeandalucia.es (R.A.C.-J.); 2 Multiprofessional Teaching Unit for Family and Community Care of the Córdoba and Guadalquivir Health District, 14011 Cordoba, Spain; 3 Group for the Evaluation and Improvement of the Program for Preventive Activities and Health Promotion (PAPPS-semFYC), 08009 Barcelona, Spain; 4 Córdoba and Guadalquivir Health District, 14011 Cordoba, Spain; 5 “Matrona Antonia Mesa Fernández” Health Center, UGC Cabra, AGS Sur de Córdoba, Cabra, 14940 Córdoba, Spain; 6 Department of Health Sciences of Burgos, University of Burgos, 09001 Burgos, Spain; jejavier@ubu.es (J.J.G.-B.); jfmielgo@ubu.es (J.M.-A.); mspelaez@ubu.es (M.S.-P.); mjgonzalez@ubu.es (J.G.-S.)

**Keywords:** COVID-19, SARS-CoV-2, predictive value, primary care, health professional

## Abstract

Background: Despite the impact that the SARS-CoV-2 virus infection has presented in Spain, data on the diagnostic capacity of the symptoms associated with this infection are limited, especially among patients with mild symptoms and who are detected in the primary care field (PC). The objective of the present study was to know the associated symptoms and their predictive criterial validity in SARS-CoV-2 infection among professionals working in PC. Methods: A cross-sectional, multicenter study was carried out in the Spanish National Health System, through an epidemiological survey directed to patients who underwent the PCR test for SARS-CoV-2 in the PC setting. Results: A total of 1612 patients participated, of which 86.6% were PC healthcare professionals, and of these, 67.4% family doctors. Hyposmia, with a sensitivity of 42.69% (95% CI: 37.30–48.08) and a specificity of 95.91% (95% CI: 94.78–97.03), and ageusia with a sensitivity of 39.47% (34.15–44.80) and a specificity of 95.20% (93.98–96.41) were the symptoms with the highest criteria validity indexes. Conclusions: This study identifies the specific symptoms of loss of smell or taste as the most frequently associated with SARS-CoV-2 infection, essential in the detection of COVID-19 given its high frequency and predictive capacity.

## 1. Introduction

In December 2019, a group of 27 cases with pneumonia of unknown origin was identified in the city of Wuhan, China [1]. Subsequently, in January 2020, the Chinese authorities identified as a possible etiological agent of the outbreak a new virus of the Coronaviridae family, which later received the name of severe acute respiratory syndrome coronavirus type 2 (SARS-CoV-2). Since then, the infection has spread to all continents, being declared a SARS-CoV-2 infection pandemic on 11 March 2020 by the World Health Organization (WHO) [2].

The symptoms associated with the infection by SARS-CoV-2 constitutes one of the topics of interest from the point of view of public health, given the explosion and rapid transmission of the virus in all continents and the appearance of new variants of it [1]. SARS CoV-2 infection can manifest asymptomatically or generate mild, moderate, or severe diverse symptoms, which can affect all organs and systems. Initially, the WHO identified fever, cough, or asthenia as symptoms of SARS-CoV-2 infection [2]. Later, other symptoms were added including olfactory or gustatory dysfunction, coagulation disorders or gastrointestinal symptoms, such as nausea or abdominal pain [3].

An international meta-analysis on the magnitude of the asymptomatic population with SARS-CoV-2 virus infection showed that the proportion of asymptomatic patients ranged from 1.4% to 78.3% [4]. In Spain, the ENE COVID study, a population-based sero-epidemiological longitudinal study carried out throughout 2020, revealed that the percentage of the asymptomatic population with SARS-CoV-2 virus infection ranged between 66.2% (first round of the study carried out in April and May) [5] and 79.4% (fourth round carried out in November) [6], identifying in the latter round 13.1% of paucisymptomatic patients; 4.7% with 3–5 symptoms and the remaining with more than five symptoms [6].

The predictive validity of the symptoms associated with the SARS CoV-2 virus infection varies depending on the prevalence of the infection. As the WHO indicates “*the probability that a person with a positive result is truly infected by this virus decreases as the prevalence decreases, regardless of the specificity of the test*” [7], hence the importance of taking into account not only the result of the diagnostic test, but also the clinical signs and symptoms, the confirmed status of the contacts, or the time of evolution of the infection. A study carried out in healthcare professionals in the Netherlands showed that general non-respiratory symptoms (muscle pain, eye pain, general malaise, headache, extreme tiredness and fever) were reported more frequently by healthcare personnel with a positive diagnostic test, and these symptoms were strongly associated with the positivity of the SARS-CoV-2 test in contrast to respiratory symptoms such as coughing and sneezing [8]. Since the clinical manifestations of patients with COVID-19 are often nonspecific especially in the initial phase [9], and resemble other similar diseases such as influenza, the clinical diagnosis of COVID-19 is really complicated [10]. For this reason, several studies have investigated possible predictive models of symptoms associated with SARS CoV-2 virus infection, obtaining heterogeneous results in the different international series [11,12,13].

Despite the impact that SARS CoV-2 virus infection has presented in Spain, data on the diagnostic capacity of the symptoms associated with this infection are limited [10,14], especially among patients with mild and that are detected in the primary care field (PC), despite the fact that they represent more than 80% of the reported cases [14].

The objective of the study was to know the associated symptoms and their predictive criterial validity in SARS-CoV-2 infection among professionals working in PC.

## 2. Materials and Methods

### 2.1. Study Design—Participants

A descriptive observational study was carried out, with a comparison control group, through an epidemiological survey directed to subjects who underwent the PCR test for the detection of SARS-CoV-2 infection in the field of PC.

The population and geographic scope of the study was the Spanish territory; on the one hand, the South Health Management Area of Córdoba and the Córdoba and Guadalquivir Health District (both institutions belonging to the Andalusian Health Service (AHS)), and on the other, the partners of the Spanish Society of Family and Community Medicine (semFYC). The study subjects were those who underwent the PCR test (real-time polymerase chain reaction [RT-PCR]), for the detection of SARS-CoV-2 during the period between 28 March and 30 September 2020.

The sample size was calculated based on the results obtained in a preliminary study [15], considering the prevalence of detected COVID-19 infection as the main parameter to estimate. Assuming that the population size is unknown, and using the calculation based on a binomial distribution, a sample with at least 1257 individuals should be selected to calculate an estimated proportion of 13.7% and a width of the confidence interval of 2%, with a confidence level of 95% (calculations made with the Winepi application: http://www.winepi.net/f102.php (accessed on 15 April 2020)).

### 2.2. Procedure—Data Collection

Two procedures for obtaining the information were used. In the first phase (subsample of the AHS) the telephone survey was used to interview all patients who were requested a sample for the diagnosis of COVID-19, while in the second phase (subsample of semFYC partners), the data were collected through a survey completed by the subject himself via online. For this purpose, semFYC partners were contacted by email, inviting them to participate, accessing the online form (https://docs.google.com/forms/d/e/1FAIpQLSeYTw5lmJnQKNN6SDp73KN5M0e7kFIFTZkOSOdDuEpKcZkUuA/viewform (accessed on 15 April 2020)).

The project was adjusted to the rules of good clinical practice (art. 34 RD 223/2004; community directive 2001/20/CE), and to the protection of personal data and confidentiality (European Data Protection Regulation, and Organic Law 3/2018 Protection of Personal Data and guarantee of digital rights). The study was authorized by the Directorate-management of the Córdoba and Guadalquivir Health District and by the Management of the South Córdoba Health Management Area and approved by the Ethics and Clinical Research Committee of the Reina Sofía Hospital in Córdoba. Informed consent was requested from all study subjects before completing the survey.

### 2.3. Main Outcomes—Instruments

A questionnaire was design to collect information related to sociodemographic variables (age, gender, area of residence—urban, if they lived in a nucleus of 20,000 inhabitants or more, semi-urban, if they lived in populations between 10,000 and 20,000 inhabitants, and rural, with less than 10,000 inhabitants-, institutionalized vs. non-institutionalized), work (health personal professional category), type of health coverage (public or private health insurance or both), contact with a sick patient with COVID-19, symptoms suffered in recent weeks (sore throat, headache, cough, nasal congestion, fever, sweating, hypothermia, dyspnea, chest pain, tiredness, joint pain, myalgia, general malaise, smell or taste disorders, aphonia, hoarseness, vomiting, nausea, stomach pain, hemoptysis, dysphonia, eye disorders, skin lesions, dizziness, vertigo, tremors, others), first symptom occurred, previous chronic pathologies (arterial hypertension, diabetes mellitus, asthma, chronic obstructive pulmonary disease, dyslipidemia, overweight/obesity, heart disease, cancer, immunodeficiencies, kidney or liver failure, depression, anxiety, cerebrovascular disease, endocrine diseases, others), tobacco use, and PCR test result for the SARS-CoV-2.

### 2.4. Statistical Analysis

A univariate statistical analysis was performed, followed by bivariate, comparing the characteristics according to the study groups using the Chi-square test or the Student’s t test (after checking the fit to a normal distribution, applying the Kolmogorov-Smirnov test). The magnitude of the association was analyzed by calculating the crude ORs and their corresponding 95% confidence intervals (95% CI). Once the variables related to the presence of positive PCR were detected, a multivariate analysis was performed, using non-conditional binary logistic regression with the calculation of the adjusted ORs. Sociodemographic, occupational, clinical manifestations and comorbidity were included in the maximum model as independent variables, and the presence or absence of COVID-19 disease as a dependent variable. The variables whose *p* value > 0.05 with the Wald statistic were eliminated from the multivariate model until obtaining the most parsimonious model. The Hosmer-Lemeshow test was used to check the fit of the model. To analyze the predictive criterial validity of the symptoms of COVID-19, the Sensitivity and Specificity parameters and the positive (PPV) and negative (NPV) predictive values, with their respective 95% CI, were calculated. Statistical analysis was carried out with the SPSS v.17.0 and EPIDAT 4.2 programs.

## 3. Results

A total of 1612 patients were evaluated, of whom 988 (61.3%) were semFYC partners and answered the online questionnaire, and 624 (38.7%) were interviewed by telephone. Table 1 shows the sociodemographic and work variables of the subjects studied. The mean age ± standard deviation was 46.0 ± 11.5 years (limits: 12–94); 71.4% were women. 86% were PA health professionals, and of these, 67.4% were family doctors.

The frequency of patients with COVID-19 was 21.2% (95% CI: 19.2%–23.3%), being 14.2% in the semFYC sample and 32.4% in the AHS sample (*p* = 0.001).

Table 2 shows the comorbidity in the study subjects. Only diabetes mellitus was more prevalent in the group of patients with SARS-Cov-2 infection than in those without it (OR = 1.89; 95% CI: 1.03–3.50; *p* = 0.037).

Table 3 shows the symptoms manifested by the patients and their association, by bivariate analysis, to the presence or absence of SARS-CoV-2 infection. Symptoms that stand out for the magnitude of their relationship with COVID-19 are the concomitant ageusia and hyposmia (OR = 23.48; 95% CI: 16.80–32.84), followed by the presence of acrosyndrome (OR = 12.72; 95% CI: 3.48–46.48), loss of appetite (OR = 4.82; 95% CI: 3.47–6.69), and the feeling of fatigue or tiredness (OR = 4.56 (3.53–5.89)). Using logistic regression (Table 4), it was found that the most strongly associated variables, independently, with the presence of SARS-CoV-2 infection in the subjects studied were: hyposmia and ageusia concomitantly (OR = 20.89; 95% CI: 11.93–36.59), acrosyndrome (OR = 11.66; 95% CI: 1.48–91.99) or feeling of fatigue or tiredness (OR = 3.15; 95% CI: 1.95–5.07).

Finally, Table 5 shows the criterial validity parameters of the symptoms related to SARS-CoV-2 infection in the subjects studied. Hyposmia, with a sensitivity of 42.69% (95% CI: 37.30–48.08) and a specificity of 95.91% (95% CI: 94.78–97.03), and ageusia, with a sensitivity of 39.47% (34.15–44.80) and a specificity of 95.20% (93.98–96.41), were the symptoms with higher criterial validity rates.

## 4. Discussion

The present study reveals the symptoms associated with SARS-CoV2 infection, as well as its predictive criterion validity in Spanish primary health care workers. Due to the magnitude of the association with SARS-CoV-2 infection, the symptoms of hyposmia and ageusia concomitantly stand out, as well as acrosyndrome, or the feeling of fatigue or tiredness. Of all the symptoms analyzed in the study, the combination of hyposmia and ageusia, with a sensitivity of 53.8% (95% CI: 48.37–59.23) and a specificity of 95.28% (95% CI: 94.07–96.48), were the symptoms with the highest criteria validity indexes.

Most of the previous studies about the symptoms of COVID-19 are primarily descriptive investigations and focus on hospitalized and high-risk subjects, skewing the information available on the most characteristic symptomatology towards people with more severe disease [16,17]. Although the evidence related to COVID-19 symptoms is highly variable, and it is difficult to find studies evaluating different combinations, loss of taste and smell have been shown to be especially sensitive symptoms in its diagnosis [17]. At least two-thirds of infected people who are not admitted to hospital describe a loss of smell and taste [16]; and although the pathogenesis of taste disorders in patients with COVID-19 is largely independent of smell, and isolated taste disorders are important in the diagnosis of COVID-19 due to their specificity, the combination of both symptoms appears to be an important determining factor in the diagnosis of the disease regardless of the classic symptoms alongside them [18].

A US-based study, which evaluated both COVID-19 positive and negative patients but with flu-like symptoms, reported loss of smell and taste in 68% and 71% of COVID-19 positive subjects and in 16% and 17% of negative patients; finding statistically significant chemosensory differences in positive cases for COVID-19 compared to negative ones [19]. In line with our results, Dixon et al. [12] found that the key symptoms to identify active SARS-CoV-2 infection were anosmia and ageusia, especially in association with fever. Also, Antonelli et al. [20] showed that loss of taste and smell correctly identified 69% and 83% of COVID-19 cases in the three- and seven-day analysis, and after adding headache and fatigue the proportion of cases of COVID-19 correctly recognized increased to 92%. Anosmia, ageusia, fatigue, persistent cough, and loss of appetite were identified by Menni et al. as the most characteristic symptoms of COVID-19 [11].

Most of the studies that consider chemosensitive dysfunctions during COVID-19 focus on the analysis of olfactory disorders and their pathogenic implication [21], neglecting taste dysfunctions that are frequently considered a consequence of postnasal olfactory loss [22]. In this sense, there is increasing scientific evidence that points to both smell and taste alterations as the main early and frequent symptoms of COVID-19 [23,24,25,26], which is supported by the findings of this research pointing to hyposmia and ageusia as the symptoms with the highest criteria validity indexes in the clinical diagnosis of COVID-19. This fact makes it easier to distinguish SARS-Cov-2 infection from other respiratory viruses, acquiring greater relevance in flu season. Paying special attention to loss of smell and taste can help healthcare professionals distinguish COVID-19 from influenza, especially in community or urgent care settings where rapid tests may be limited.

The study has limitations that must be considered. In the first place, it is convenient to indicate that probabilistic sampling techniques were not used, but rather that the possibility of participating in the study was offered to the group of subjects that constituted the study subpopulations (the health professionals of the SAS, and the partners of the semFYC scientific society). As in all studies conducted through surveys, the degree of interest and motivation for the subject of the study subjects can lead to a selection bias to a greater or lesser extent. Despite this, we consider that with the sample size reached, the possibility of the sample being representative of the study population is high, to which we must add that the questionnaire was answered anonymously, facilitating the veracity of the responses and therefore, the validity of the results by minimizing the risk of information biases. It is also necessary to take into account the possible existence of confounding factors, which were controlled by multivariate analysis.

Regarding the strengths, it is worth highlighting the sample size of the study, being one of the investigations carried out with the largest number of health professionals in the primary health care sector in Spain. On the other hand, by encompassing patients from the community, mostly not hospitalized (who would be the most serious and with the worst prognosis), the external validity of the study is reinforced.

## 5. Conclusions

The present study reveals the symptoms associated with SARS-CoV-2 infection, as well as its predictive criterion validity in Spanish primary health care workers. Of all the symptoms associated with SARS-CoV-2 infection, the combination of hyposmia and ageusia were the symptoms with the highest criteria validity indexes. Therefore, these symptoms should be taken into account when assessing the presence of SARS CoV-2 infection, given its high frequency and predictive capacity.

## Figures and Tables

**Table 1:** Sociodemographic characteristics of the study subjects.

Characteristics	Total Sample	SARS-CoV-2 Infection		*p* Value
	(n = 1612)	Yes (n = 342)	No (n = 1270)	
	n (%)	n (%)	n (%)	
Age				
≤37 years old	433 (26.9)	84 (19.4)	349 (80.6)	
38 to 47 years old	384 (23.8)	85 (22.1)	299 (77.9)	0.556
48 to 57 years old	493 (30.6)	102 (20.7)	391 (79.3)	
≥58 years old	302 (18.7)	71 (23.5)	231 (76.5)	
Gender				
Male	461 (28.6)	106 (23.4)	353 (76.6)	0.169
Female	1151 (71.4)	234 (20.3)	917 (79.7)	
Area of residence (inhabitants)				
Rural (<10,000)	292 (18.1)	52 (17.8)	240 (82.2)	
Semi-urban (10,000–20,000)	787 (48.8)	120 (15.2)	667 (84.8)	<0.001
Urban (>20,000)	533 (33.1)	170 (31.9)	363 (68.1)	
Type of work				
Health work	1396 (86.6)	281 (20.1)	1115 (79.9)	0.007
Nonhealth work	216 (13.4)	61 (28.2)	115 (71.8)	
Profession				
Family doctor	1086 (67.4)	163 (15.0)	923 (85.0)	
Nurse	148 (9.2)	52 (35.1)	96 (64.9)	<0.001
Nursing assistant	105 (6.5)	44 (12.9)	61 (58.0)	
Warden	18 (1.1)	3 (16.7)	15 (83.3)	
Administrative	25 (1.6)	11 (44.0)	14 (56.0)	
Other	230 (14.3)	69 (30.0)	161 (70.0)	
Type of health coverage				
Public	1388 (86.1)	281 (20.2)	1107 (79.8)	0.046
Private	47 (2.9)	11 (23.4)	36 (7.6)	
Both	177 (11.0)	50 (28.2)	127 (71.8)	

**Table 2:** Comorbidity in patients with or without SARS-CoV2 infection.

Comorbidity	Total Sample	SARS-CoV-2 Infection		OR (IC 95%)	*p* Value
	(n = 1612)	Yes (n = 342)	No (n = 1270)		
	n (%)	n (%)	n (%)		
Arterial hypertension	167 (10.4)	43 (25.7)	124 (74.3)	1.33 (0.92–1.92)	0.130
Diabetes Mellitus	48 (3.0)	16 (33.3)	32 (66.7)	1.89 (1.03–3.50)	*0.037*
Dyslipidemia	136 (8.4)	34 (25.0)	102 (75.0)	1.26 (0.84–1.90)	0.259
Overweight/obesity	269 (16.7)	63 (23.4)	206 (76.6)	1.17 (0.85–1.59)	0.333
Asthma	143 (8.9)	31 (21.7)	112 (78.3)	1.03 (0.68–1.56)	0.887
COPD	18 (1.1)	5 (27.8)	13 (72.2)	1.43 (0.51–4.05)	0.493
Heart disease	36 (2.2)	8 (22.2)	28 (77.8)	1.06 (0.48–2.35)	0.881
Endocrine disease	103 (6.4)	28 (27.2)	75 (72.8)	1.42 (0.90–2.23)	0.126
Cancer	24 (1.5)	6 (25.0)	18 (75.0)	1.24 (0.49–3.15)	0.648

OR = Odds Ratio; 95% CI = 95% confidence interval; SARS-CoV-2 = Severe Acute Respiratory Syndrome Coronavirus type 2; COPD = Chronic obstructive pulmonary disease. *p* values obtained by the Chi-square test.

**Table 3:** Clinical manifestations in study subjects.

Manifestations	Total Sample	SARS-CoV-2 Infection		OR (CI 95%)	*p* Value
	(n = 1612)	Yes (n = 342)	No (n = 1270)		
	n (%)	n (%)	n (%)		
General and non-specific					
General discomfort	361 (22.4)	135 (37.4)	226 (62.6)	3.01 (2.32–3.91)	<0.001
Fever	292 (18.1)	121 (41.4)	171 (58.6)	3.52 (2.67–4.63)	<0.001
Muscle pain	393 (24.4)	169 (43.0)	224 (57.0)	4.56 (3.53–5.89)	<0.001
Dizziness	47 (2.9)	15 (31.9)	32 (68.1)	1.77 (0.95–3.32)	0.069
Chills	187 (11.6)	57 (30.5)	130 (69.5)	1.75 (1.25–2.46)	0.001
Sweating	107 (6.6)	33 (30.8)	74 (69.2)	1.73 (1.12–2.65)	0.012
Hypothermia	76 (4.7)	24 (31.6)	52 (68.4)	1.77 (1.07–2.91)	0.024
Apetite loss	171 (10.6)	87 (50.9)	84 (49.1)	4.82 (3.47–6.69)	<0.001
Respiratory					
Cough	443 (27.5)	146 (33.0)	297 (67.0)	2.44 (1.89–3.13)	<0.001
Pharyngeal pain	365 (22.6)	91 (24.9)	274 (75.1)	1.32 (1.00–1.73)	0.048
Nasal congestion	243 (15.1)	80 (32.9)	163 (67.1)	2.07 (1.54–2.80)	<0.001
Spits	43 (2.7)	14 (32.6)	29 (67.4)	1.83 (0.95–3.50)	0.065
Respiratory difficulty	141 (8.7)	51 (36.2)	90 (63.8)	2.40 (1.60–3.59)	<0.001
Digestive					
Nausea	69 (4.3)	29 (42.0)	40 (58.0)	2.85 (1.74–4.67)	<0.001
Vomiting	38 (2.4)	12 (31.6)	26 (68.4)	1.74 (0.87–3.48)	0.114
Abdominal pain	62 (3.8)	27 (43.5)	35 (56.5)	3.02 (1.80–5.07)	<0.001
Diarrhea	209 (13.0)	71 (34.0)	138 (66.0)	2.15 (1.57–2.95)	<0.001
Otolaryngological					
Dysphonia	105 (6.5)	34 (32.4)	71 (67.6)	1.86 (1.22–2.86)	0.004
Hoarseness	96 (6.0)	29 (30.2)	67 (69.8)	1.66 (1.06–2.62)	0.026
Ageusia	196 (12.2)	135 (68.9)	61 (31.1)	12.93 (9.23–18.09)	<0.001
Hyposmia	198 (12.3)	146 (73.7)	52 (26.3)	17.45 (12.29–24.77)	<0.001
Ageusia and hyposmia	244 (15.1)	184 (75.4)	60 (24.6)	23.48 (16.80–32.84)	<0.001
Cardiological					
Thoracic oppression	75 (4.7)	27 (31.8)	58 (68.2)	1.92 (1.17–3.16)	0.009
Neurological					
Headache	506 (31.4)	159 (31.4)	347 (68.6)	2.31 (1.81–2.95)	<0.001
Ophthalmic					
Ophthalmic	175 (10.9)	69 (39.4)	106 (60.6)	2.77 (1.99–3.86)	<0.001
Dermatological					
Facial erythema	18 (1.1)	4 (22.2)	14 (77.8)	1.06 (0.35–3.25)	0.916
Acrosyndrome	13 (0.8)	10 (76.9)	3 (23.1)	12.72 (3.48–46.48)	<0.001

OR = Odds Ratio; 95 CI = 95% Confidence Interval; SARS-CoV-2 = Severe Acute Respiratory Syndrome Coronavirus type 2.

**Table 4:** Factors associated with SARS-CoV-2 infection by multivariate analysis (binary logistic regression).

Associated Factors	OR	CI 95%	*p* Value
**Profession**			
Family doctor	(Ref.)	(Ref.)	(Ref.)
Nurse	2.50	1.50–4.17	0.004
Nursing assistant	3.13	1.74–5.62	0.001
Warden	0.19	0.03–1.23	0.189
Administrative	4.05	1.19–13.76	0.025
Other	2.56	1.27–5.17	0.009
Close contact with sick (COVID19) person	2.18	1.55–3.07	<0.001
Tiredness or fatigue	3.15	1.95–5.07	<0.001
Dizziness	2.64	1.08–6.45	0.033
Nasal congestion	1.84	1.10–3.06	0.020
Sweating	2.03	1.04–3.95	0.037
Pharyngeal pain	1.90	1.23–2.93	0.004
Hyposmia	2.89	1.33–6.27	0.007
Ageusia	2.55	1.12–5.83	0.026
Hyposmia and ageusia	20.89	11.93–36.59	<0.001
Acrosyndrome	11.66	1.48–91.99	0.020

OR = Odds Ratio; 95% CI = 95% Confidence Interval; (Ref.): Reference category; SARS- CoV-2 = Severe Acute Respiratory Syndrome Coronavirus type 2.

**Table 5:** Sensitivity, Specificity and Predictive Values of Symptoms Associated with SARS-CoV-2 Virus Infection.

Manifestations	Se (IC 95%)	Sp (IC 95%)	VPP (IC 95%)	VPN (IC 95%)	CP+ (IC 95%)	CP- (IC 95%)
**General and non-specific**						
General discomfort	39.47 (34.15–44.80)	82.20 (80.06–84.35)	37.40 (32.27–42.53)	83.45 (81.35–85.55)	2.22 (1.86–2.68)	0.74 (0.67–0.81)
Fever	35.38 (30.17–40.59)	86.54 (84.62–88.45)	41.44 (35.62–47.26)	83.26 (81.21–85.31)	2.63 (2.15–3.21)	0.75 (0.69–0.81)
Muscle pain	49.42 (43.97–54.86)	82.36 (80.23–84.50)	43.00 (37.98–48.02)	85.81 (83.81–87.81)	2.80 (2.39–3.29)	0.61 (0.55–0.68)
Dizziness	4.39 (2.07–6.70)	97.48 (96.58–98.38)	31.91 (17.52–46.31)	79.11 (77.06–81.15)	1.74 (0.95–3.18)	0.98 (0.96–1.01)
Chills	16.67 (12.57–20.76)	89.76 (88.06–81.47)	30.48 (23.62–37.25)	80.00 (77.89–82.11)	1.63 (1.22–2.17)	0.93 (0.88–0.98)
Sweating	9.65 (6.37–12.92)	94.17 (92.85–95.50)	30.84 (21.62–40.06)	79.47 (77.39–81.54)	1.66 (1.12–2.45)	0..96 (0.92–1.00)
Hypothermia	7.02 (4.16–9.87)	95.91 (94.78–97.03)	31.58 (20.47–42.69)	79.30 (77.24–81.36)	1.71 (1.07–2.74)	0.97 (0.94–1.00)
Apetite loss	25.44 (20.68–30.20)	93.39 (91.98–94.79)	50.88 (43.09–58.66)	82.30 (80.30–84.31)	3.85 (2.92–5.06)	0.80 (0.75–0.85)
**Respiratory**						
Cough	42.69 (37.30–48.08)	76.61 (74.25–78.98)	32.96 (28.47–37.45)	83.23 (81.05–85.42)	1.83 (1.56–2.14)	0.75 (0.68–0.82)
Pharyngeal pain	26.0 (21.78–31.44)	78.43 (76.12–80.73)	24.93 (20.36–29.51)	79.87 (77.61–82.14)	1.23 (1.00–1.51)	0.94 (0.87–1.00)
Nasal congestion	23.39 (18.76–28.02)	87.17 (85.29–89.04)	32.92 (26.81–39.04)	80.86 (78.74–82.98)	1.82 (1.43–2.32)	0.88 (0.83–0.94)
Spits	4.09 (1.85–6.34)	97.72 (96.86–98.58)	32.56 (17.39–47.73)	79.09 (77.05–81.14)	1.79 (0.96–3.35)	0.98 (0.96–1.00)
Respiratory difficulty	12.28 (8.66–15.91)	94.49 (93.19–95.78)	37.50 (28.09–46.91)	80.00 (77.94–82.06)	2.23 (1.55–3.20)	0.93 (0.89–0.97)
**Digestive**						
Nausea	8.48 (5.38–11.58)	96.85 (95.85–97.85)	42.03 (29.66–54.40)	79.71 (77.68–81.75)	2.70 (1.70–4.29)	0.94 (0.90–1.00)
Vomiting	3.51 (1.41–5.61)	97.95 (97.13–98.77)	31.58 (15.48–47.67)	79.03 (76.99–81.08)	1.71 (0.87–3.36)	0.99 (0.96–1.01)
Abdominal pain	7.89 (4.89–10.90)	97.24 (96.30–98.18)	43.55 (30.40–56.70)	79.68 (77.64–81.71)	2.86 (1.76–4.66)	0.95 (0.92–0.98)
Diarrhea	20.76 (16.32–25.20)	89.13 (87.38–90.88)	33.97 (27.31–40.63)	80.68 (78.58–82.79)	1.91 (1.47–2.48)	0.89 (0.84–0.94)
**Otolaryngological**						
Dysphonia	9.94 (6.62–13.26)	94.41 (93.11–95.71)	32.38 (22.95–41.81)	79.56 (77.49–81.63)	1.78 (1.20–2.63)	0.95 (0.92–0.99)
Hoarseness	8.48 (5.38–11.58)	94.72 (93.46–95.99)	30.21 (20.50–39.91)	79.35 (77.28–81.42)	1.61 (1.06–2.44)	0.97 (0.93–1.00)
Ageusia	39.47 (34.15–44.80)	95.20 (93.98–96.41)	68.88 (62.14–75.61)	85.38 (83.51–87.26)	8.22 (6.22–10.85)	0.64 (0.58–0.69)
Hyposmia	42.69 (37.30–48.08)	95.91 (94.78–97.03)	73.74 (67.36–80.12)	86.14 (84.30–87.90)	10.43 (7.78–13.98)	0.60 (0.54–0.66)
Ageusia and hyposmia	24.59 (18.98–30.20)	11.55 (9.82–13.28)	4.72 (3.52–5.93)	46.20 (40.77–51.63)	0.28 (0.22–0.35)	6.53 (5.55–7.69)
**Cardiological**						
Thoracic oppression	7.89 (4.89–10.90)	95.43 (94.43–96.62)	31.76 (21.28–42.25)	79.37 (77.31–81.43)	1.73 (1.11–2.69)	0.97 (0.93–1.00)
**Neurological manifestations**						
Headache	49.49 (41.06–51.92)	72.68 (70.19–75.17)	31.42 (27.28–35.57)	83.45 (81.22–85.69)	1.70 (1.47–1.97)	0.74 (0.66–0.82)
**Ophthalmic**						
Ophthalmic	20.18 (15.78–24.57)	91.65 (90.09–93.21)	39.43 (31.90–46.95)	81.00 (78.94–83.07)	2.42 (1.83–3.19)	0.87 (0.82–0.92)
**Dermatological**						
Facial erythema	1.17 (0.00–2.46)	98.90 (98.28–99.51)	22.22 (0.24–44.21)	78.80 (76.76–80.23)	1.06 (0.35–3.20)	1.00 (0.99–1.01)
Acrosyndrome	2.92 (0.99–4.86)	99.76 (99.46–100)	76.92 (50.17–100)	79.24 (77.22–81.26)	12.38 (3.43–44.73)	0.97 (0.96–0.99)

Se: sensitivity; Sp: specificity; 95% CI: 95% confidence interval; NPV negative predictive value; PP: positive predictive value; CP +: positive likelihood ratio; CP−: negative likelihood ratio.
